# The nutritive value of marula (*Sclerocarya birrea*) seed cake for broiler chickens: nutritional composition, performance, carcass characteristics and oxidative and mycotoxin status

**DOI:** 10.1007/s11250-017-1269-9

**Published:** 2017-03-22

**Authors:** Doctor Mziwenkosi Nhlanhla Mthiyane, Bhekumusa Sabelo Mhlanga

**Affiliations:** 1 Department of Animal Science, Luyengo Campus, Faculty of Agriculture, University of Swaziland, P.O. Box M205, Luyengo, Swaziland; 2 Crane Feeds (Pty) Ltd., P.O. Box 1974, Matsapha, Swaziland

**Keywords:** Marula seed cake, Broilers, Chemical composition, Performance, Carcass characteristics, Mycotoxins

## Abstract

This study was aimed at investigating the nutritive value of marula seed cake (MSC) as an alternative protein source for broilers. In a completely randomised design involving six replicate pens of five chickens assigned to each of five treatments, body weight gain (BWG), feed intake (FI), feed conversion efficiency (FCE) and carcass characteristics were measured in an experiment in which 150 28-day-old broilers were fed maize-based diets containing, respectively, 0, 5, 10, 15 and 20% MSC at finisher phase. The results showed MSC to be remarkably high in CP (470.0 g/kg DM) and EE (343.5 g/kg DM), with moderate CF (58.2 g/kg DM), ash (54.3 g/kg DM), Ca (1.1 g/kg DM) and P (11.0 g/kg DM). Whilst very poor in lysine, MSC was found to be rich in methionine, cyst(e)ine, arginine and glutamic acid; it also contains good levels of valine, glycine, threonine, isoleucine, leucine, histidine, phenylalanine, serine, proline and alanine. Also, it contained 85.24% oleic (OA), 9.65% palmitic and 5.11% stearic acids, with a high peroxide value and low levels of mycotoxins deoxynivalenol (DON) and T-2 toxin. BWG, FI and FCE of broiler chickens significantly decreased (*P* < 0.001) as the dietary level of MSC increased. Further, dietary MSC significantly decreased bird live weight at slaughter (*P* < 0.001), plucked weight (*P* < 0.001), dressed weight (*P* < 0.001) and weights of the liver (*P* < 0.001) and neck (*P* < 0.05). The results therefore demonstrate MSC to be a good source of CP, fat, Ca, P, amino acids (except lysine) and OA that can replace soya bean meal (SBM) in broiler diets. However, its use is currently limited by lipid peroxidation and presence of mycotoxins.

## Introduction

The rapidly increasing human population particularly in the developing world has increased the demand for animal protein, especially for pork and poultry products, for human nutrition (World Bank Group 2015). This is an opportunity for smallholder farmers in Africa to increase household income and improve their livelihoods by connecting with the livestock and poultry value chain. Poultry production, in particular, has a potentially great role to play in terms of poverty alleviation in most developing countries. In Swaziland, for example, poultry are the most widely owned farm animal species, with more than 90% of rural households rearing poultry in the country (Rajiur Rahman 2012; Siyaya and Masuku 2013). However, smallholder farmers in sub-Saharan Africa (SSA) often lack access to good quality feed with sufficient energy and a balanced amino acid profile that is needed to ensure satisfactory animal performance (Martens et al. 2012).

SBM is a widely used feed supplement with high energy content and an amino acid profile close to ideal. The protein quality of SBM is high for poultry, and SBM is a particularly good source of lysine, arginine and tryptophan, but it is deficient in methionine plus cysteine, threonine and valine (Baker 2000). Globally, however, SBM is predominantly used in highly industrialised production systems for pigs and poultry, and there is little surplus SBM for smallholder farmers. Thus, many livestock farmers in most developing countries cannot afford the exorbitant costs of importation of this protein supplement. Therefore, to meet the nutritional requirements of poultry in SSA, there is a need to identify alternative low-cost protein sources (Martens et al. 2012; Acheampong-Boateng et al. 2016).

MSC is a by-product of oil extraction from the dry seeds of the ripe fruits of marula (*Sclerocarya birrea* A. Rich.), an indigenous fruit tree distributed throughout most of SSA from 17° 15′ N in the Aïr Mountains of Niger to 31° 00′ S near Port Shepstone in South Africa (Hall et al. 2002; Chirwa and Akinnifesi 2008). It is a novel locally available low-cost alternative protein supplement for livestock and poultry in Swaziland. In previous studies, it has been found to be a good dietary protein supplement for beef cattle (Mlambo et al. 2011a), dairy cattle (Mdziniso et al. 2016) and goats (Mlambo et al. 2011b). However, its nutritional value for poultry has hitherto not been investigated. This study was therefore undertaken to investigate the nutritional composition and oxidative and mycotoxin status of MSC and performance and carcass characteristics of broilers fed maize-based diets supplemented with graded levels of MSC at finisher phase.

## Materials and methods

### Location of study and source of materials

The study was conducted in a small-scale commercial broiler house outside Luve, a small town in central Swaziland that is located on the MR5 route between Mpisi and Mliba, 25 km northeast of Manzini city. The broiler house was made of wooden materials with a metal roof of corrugated iron sheets and removable tarpaulin side curtains.

All the feed materials, including MSC, as well as day-old broiler chicks, were supplied by Crane Feeds (Pty) Ltd. (Swaziland). MSC was purchased from Swazi Secrets, a local small-scale marula oil extracting firm at Mpaka (Swaziland). The oil extraction procedure has previously been described by Mlambo et al. (2011a). The unsexed day-old chicks of the Cobb 500 strain were purchased from National Chicks Swaziland at 1 day of age.

### Chemical assay

Chemical analyses of MSC samples were performed at the Central Analytical Laboratories (Roodepoort, South Africa) and AminoLab at Evonik Africa (Pty) Ltd. (South Africa). The DM (930.15), ash (942.05), CP (954.01), EE (920.39) and CF were determined according to procedures of the AOAC (2000). CP was calculated using N × 6.25. Ca and P composition was determined according to VDLUFA (2007). Amino acid analysis, preceded by acid hydrolysis, were performed (method 982.30) using an amino acid analyser (SY-KAM, Erising, Germany) following modifications by Mills et al. (1989). Analysis of fats and fatty acids was performed using a gas chromatography following the revised (method 996.06) procedures of the AOAC (2002). Mycotoxin analysis was performed using high pressure liquid chromatography-mass spectrophotometry (HPLC-MS) and the Vicam Myco6in1® LC/MS/MS method. Peroxide values were determined using the Titrino apparatus following the American Oil Chemist Society (AOCS) official method (AOAC 1993). Lipid peroxidation was determined by measuring the formation of thiobarbituric acid reactive substances (TBARS)/malondialdehyde using an HPLC method adapted from Moselhy et al. (2013).

### Experimental design, birds and diets

A total of 150 Cobb-500 28-day-old broiler chickens, fed a maize and soya bean-based commercial starter diet until they were 28 days old, were used. On day 29, they were assigned to stainless steel pens (each with five birds) so that the initial body weights (8.0 kg/pen) were equal among treatments. A completely randomised design was used with six replicate pens of five chickens assigned to each of five dietary treatments. The chickens were maintained on natural light during the day and on continuous artificial light at night. They were allowed ad libitum access to feed and water from day 29 to day 44 and were inspected daily for any health-related problems.

Iso-energetic and iso-nitrogenous maize and SBM-based diets, in mash form formulated to meet the nutritional requirements of finisher birds as recommended by the NRC (1994) and to which graded levels of MSC were sequentially added in replacement of SBM (Table 1), were used. MSC was incorporated into the diets to produce dietary treatments containing, respectively, 0 (control), 5, 10, 15 and 20% MSC.

**Table 1:** Ingredients and nutrient composition (g/kg) of experimental finisher diets (as-fed basis)

	Dietary MSC level (%)				
Ingredient	0	5	10	15	20
Yellow maize meal	639	676	684	656	597
Soya bean meal (47% CP)	190	112	74	23	−
Full-fat soya bean	64.3	100	22	−	−
MSC	−	50	100	150	200
Wheat bran	−	−	−	−	96.5
Sunflower seed meal	29.6	−	61	125	64
Fishmeal (65% CP)	−	20	20	−	−
Limestone	14.2	15	15	18	17.5
Monocalcium phosphate	5.3	11	10	11.2	10
Mineral/vitamin premix^a^	3.5	3.5	3.5	3.5	3.5
Soya bean oil	29.6	3.2	−	−	−
Salt	3.3	2.9	2.9	3.4	3.3
Lysine	2.6	2.4	3.4	4.7	4.6
Sodium bicarbonate	2.0	2.0	2.0	2.0	2.0
DL-Methionine	2.5	1.9	1.6	1.4	1.4
Threonine	0.7	0.6	0.7	0.8	0.8
Calculated composition					
ME (MJ/kg)	12.5	12.5	12.5	12.5	12.5
CP	18.0	18.0	18.0	18.0	18.0
CF	3.0	3.0	4.0	5.3	5.0
EE	6.6	6.6	6.8	8.0	9.7
Ash	4.2	4.2	4.3	4.4	4.5
NFE	56.6	56.6	54.9	51.9	50.5
Ca	0.85	0.85	0.85	0.90	0.85
P	0.64	0.64	0.68	0.73	0.76
Na (mg/kg)	0.19	0.19	0.19	0.19	0.19
Cl (mg/kg)	0.25	0.25	0.25	0.25	0.24
K (mg/kg)	0.77	0.77	0.72	0.71	0.75
Lysine	1.1	1.1	1.1	1.08	1.1
Methionine	0.50	0.50	0.49	0.47	0.46
Cysteine	0.32	0.32	0.34	0.37	0.39
Methionine + cysteine	0.82	0.82	0.83	0.84	0.85
Threonine	0.74	0.74	0.75	0.76	0.77
Tryptophan	0.21	0.21	0.20	0.21	0.21
Valine	0.02	0.02	0	0	0
Arginine	1.30	1.30	1.48	1.69	1.85
Isoleucine	0.72	0.72	0.69	0.66	0.63
Xantophyll (ppm)	13.5	13.5	13.7	13.1	11.9

^a^The mineral/vitamin premix supplied the following per kilogram of complete feed: vitamin A (retinol acetate), 2250 mg; vitamin D3 (cholecalciferol), 82.5 mg; vitamin E (d-α-tocopherol), 40,000 mg; vitamin K3 (menadione), 1300 mg; vitamin B1 (thiamine mononitrate), 1300 mg; vitamin B2 (riboflavin), 3300 mg; vitamin B3 (nicotinic acid), 33,000 mg; calcium panthonate, 8000 mg; vitamin B6 (pyridoxine chlorhydrate), 2000 mg; folic acid, 1300 mg; vitamin B12 (cyanocobalamin), 6.5 mg; biotin, 70 mg; antioxidant, 125,000 mg; Mn, 70,000 mg; Fe, 25,000 mg; Zn, 70,000 mg; Cu, 6500 mg; Co, 350 mg; I, 1300 mg; Se, 200 mg; choline, 200,000 mg

### Measurements

Chickens were group weighed by pen at the beginning (day 29) and then daily thereafter until the end of the experiment on day 44. Also, feed offered and left was weighed per pen daily in order to calculate average FI per bird per day. Feed conversion ratio was calculated from average BWG per bird per day (g) divided by average FI per bird per day (g).

On day 44, all the birds were weighed again in order to determine live weight at slaughter and then killed by stunning with electric shock and slitting the throat of each bird with a sharp knife. After plucking off the feathers, the carcasses were weighed in order to determine plucked weights. Carcasses were then dressed and weighed in order to determine dressed weight. During evisceration, the internal organs were removed and weighed.

### Statistical analyses

Data on growth performance and carcass/organ characteristics of birds were analysed using the GLM procedure of Minitab (2000). Statistical significance was accepted based on the 0.05 level of probability.

## Results and discussion

Our results showed MSC to have a remarkably high content of CP (470.0 g/kg DM) and EE (343.5 g/kg DM), with moderate CF (58.2 g/kg DM) and ash (54.3 g/kg DM) contents (Table 2). The results corroborate previous findings by Mlambo et al. (2011a) and Mdziniso et al. (2016) who reported similar CP (470.0–470.21 g/kg DM), EE (289.6–394 g/kg DM) and ash (55.5–66.1 g/kg DM) values in MSC. The CP content of MSC evidently surpasses that of most alternative protein sources for non-ruminants such as sunflower oilcake (341 g/kg DM), canola oilcake (339 g/kg DM), cotton seed cake (403 g/kg DM) and tropical leguminous forages (≤360 g/kg DM) (Ramachandran et al. 2007; Martens et al. 2012). In this connection, MSC is indeed a good source of CP at par with SBM (Banaszkiewicz 2011).

**Table 2:** The proximate, mineral and amino acid composition of MSC

Component	Amount
DM (g/kg)	946.6
CP (g/kg DM)	470.0
EE (g/kg DM)	343.5
CF (g/kg DM)	58.2
Ash (g/kg DM)	54.3
Ca (g/kg DM)	1.1
P (g/kg DM)	11.0
Na (g/kg DM)	ND
Amino acids (% of DM)	
Lysine	0.88
Methionine	0.79
Cystine	1.18
Methionine + cystine	1.96
Threonine	0.98
Isoleucine	1.77
Leucine	2.66
Phenylalanine	1.91
Histidine	1.13
Arginine	6.36
Valine	2.14
Alanine	1.40
Aspartic acid	3.51
Glycine	2.11
Serine	1.91
Proline	1.44
Glutamic acid	10.78
Total (without ammonia)	41.23
Ammonia	1.11
Total	42.34

*ND* not detected

Also, whilst very poor in lysine, MSC was found to be rich in sulphur amino acids methionine and cyst(e)ine, with an abundance of arginine and glutamic acid (Table 2). It was also found to contain rather good levels of valine, glycine, threonine, isoleucine, leucine, histidine, phenylalanine, serine, proline and alanine that are similar to typical values for SBM (Goldflus et al. 2006; Carrera et al. 2011; Nahashon and Kilonzo-Nthenge 2011). These findings are in agreement with previous observations that the protein in the kernel of marula seed is limiting in lysine yet a good source of leucine and sulphur-containing amino acids methionine and cysteine (Mojeremane and Tshwenyane 2004; Mariod et al. 2005; Muhammad et al. 2011a), with a predominance of glutamic acid and arginine (Mariod and Abdelwahab 2012). The results suggest that MSC can be used to supply much needed protein and amino acids in resource-poor farming systems in developing countries. Also, formulation of broiler diets involving MSC as a protein source in the future would not necessarily require any fortification with synthetic amino acids except for lysine.

The EE content of MSC (343.5 g/kg DM) is too high relative to other conventional protein sources such as SBM (155–247 g/kg DM; Banaszkiewicz 2011). However, this level is now rather lower than what has been previously reported (394 g/kg DM; Mlambo et al. 2011a) and is an indication that the oil extraction process at the firm that produces the MSC has improved over the years.

The mineral analysis showed the Ca content of MSC (Table 2) to be 4× lower than values previously found in marula seed kernels in Kenya (Wairagu et al. 2013). However, it was interestingly comparable to that of SBM (1.3 g/kg DM; Ramachandran et al. 2007). On the other hand, the P content of MSC was found to be approximately 7× higher than that previously found in marula seed kernels in Kenya (Wairagu et al. 2013), probably due to sub-species or genetic variation (Moganedi et al. 2011; Nyoka et al. 2015). A comparable value of P in the seed kernel (9.0 g/kg DM) has previously been observed by Akinnifesi et al. (2008). However, of great interest is that the P content of MSC was found to be 1.6× higher than that of SBM (6.9 g/kg DM; Ramachandran et al. 2007).

In terms of fats and fatty acid composition, MSC was found to contain 85.24% monounsaturated (MUFAs) and 14.76% saturated (SFAs) fatty acids, with neither polyunsaturated (PUFAs) nor trans (TFAs) fatty acids. Interestingly, all the MUFAs were composed of OA, a fatty acid less sensitive to oxidation relative to PUFAs, whilst the SFAs were only palmitic (9.65%) and stearic (5.11%) acids (Table 3). In previous studies, the oil in marula seed kernels has been reported to be 10× more stable to oxidation and to contain a similar fatty acid composition to olive oil (Mariod and Abdelwahab 2012), with as high as 67.2% OA, 5.9% linoleic acid, 14.1% palmitic acid and traces of linolenic acid (Mariod 2005). Hence, we suspect that lipid peroxidation might have occurred resulting in the depletion of PUFAs that might have been present in the MSC used in our study. Therefore, with such a high MUFA content in the form of OA, and if it can be protected from lipid peroxidation during processing and storage thereafter, MSC incorporation into broiler diets would be expected to increase the level of MUFAs, and possibly PUFAs, in the meat and, consequently, improve health benefits to consumers. Considering the hypocholesterolemic properties of OA (Kurushima et al. 1995), a substantial increase in the content of this fatty acid in broiler meat is highly desirable. Also, with the oil in marula seed kernels being highly stable to oxidation, it is further hypothesised that MSC incorporation into broiler diets would enhance the shelf life of meat.

**Table 3:** Total fats and fatty acid content of MSC

Fats (% of total)	
Monounsaturated fats	85.24
Polyunsaturated fats	0.00
Saturated fats	14.76
Trans fatty acids	0.00
Fatty acids (g/100 g)	
C16:0 (palmitic acid)	9.65
C18:0 (stearic acid)	5.11
C18:1 cis-9 (oleic acid)	85.24

Whilst MSC is evidently a good protein, energy, mineral, amino acid and OA source for poultry in terms of its nutritional composition, its dietary substitution for SBM appears to cause remarkably deleterious effects on broiler performance. In this connection, BWG, FI and FCE were significantly decreased (*P* < 0.001) by dietary inclusion of MSC (Table 4). Similar depressive effects of MSC on FI, BWG and FCE in broilers have previously been observed at our university research farm (Magagula 2013; Mamba 2013). Also, a significant decrease in body weights in contrast to an increase in serum total protein, albumin, bilirubin, transaminases, creatinine, urea, uric acid and electrolytes was observed in rats orally administered with 3000 and 4000 mg/kg body weight of marula fruit kernel extract (Muhammad et al. 2011b).

**Table 4:** Body weight gain, feed intake and feed conversion efficiency in broilers fed diets containing graded levels of MSC at finisher phase

	MSC dietary level (%)						
Items	0	5	10	15	20	SEM^a^	*P* value
BWG (g/bird/day)	81.33	62.90	64.52	37.05	36.87	3.295	*P* < 0.001
FI (g/bird/day)	183.17	147.50	146.50	135.30	120.0	1.911	*P* < 0.001
G/F (g/g)	0.44	0.43	0.44	0.27	0.31	0.012	*P* < 0.001

*G/F* gain/feed ratio

^a^Standard error of the mean (SEM) based on pooled estimate of variation (*n* = 6)

The deleterious effects of MSC on broiler performance were also mirrored in the live weights as well as the carcass characteristics of the birds at slaughter. In this connection, a significant decrease was observed in the live weight (*P* < 0.001), plucked weight (*P* < 0.001), dressed weight (*P* < 0.001), liver weight (*P* < 0.001) and neck weight (*P* < 0.05) of broilers as the level of MSC increased in the diet (Table 5). Although the trend was generally similar to the above carcass characteristics, there were however no significant effects (*P* > 0.05) of dietary MSC on the weights of the gizzard, heart, intestine, head, abdominal fat, feet and collective viscera. In a previous study at our institution’s research farm, Magagula (2013) also observed that dietary MSC supplementation significantly decreased the live weight, dressed weight, dressing percentage, length of feathers, as well as weights of the wing, breast, thigh, drumstick, shank, abdominal fat, liver, gizzard, heart and intestines in broilers.

**Table 5:** Carcass characteristics (g) of broilers fed diets containing graded levels of MSC at finisher phase

	MSC dietary level (%)						
Items	0	5	10	15	20	SEM^a^	*P* value
Live weight	2838.50	2482.50	2557.80	2171.00	2169.70	63.44	*P* < 0.001
Plucked weight	2439.80	2056.20	2156.00	1842.80	1844.80	69.60	*P* < 0.001
Dressed weight	1948.40	1608.00	1735.40	1451.80	1418.10	64.14	*P* < 0.001
Liver	48.73	43.67	45.83	38.00	32.33	2.52	*P* < 0.001
Gizzard	35.20	35.50	36.17	34.33	36.77	1.45	*P* > 0.05
Heart	13.43	10.83	14.50	10.33	10.53	1.45	*P* > 0.05
Intestine	103.47	106.67	113.03	99.67	99.83	8.15	*P* > 0.05
Neck	58.37	51.33	53.10	47.50	50.17	2.31	*P* < 0.05
Head	48.73	45.67	48.00	47.50	46.50	1.62	*P* > 0.05
Abdominal fat	27.93	25.50	24.87	19.67	29.03	5.12	*P* > 0.05
Feet	85.83	75.50	84.37	75.33	78.27	3.54	*P* > 0.05
Viscera	420.33	400.17	419.03	370.00	379.50	14.51	*P* > 0.05

^a^Standard error of the mean (SEM) based on pooled estimate of variation (*n* = 6)

The negative effects of MSC on performance and carcass parameters are unlikely to be due to poor palatability of MSC as marula kernels are generally consumed and cherished by people in rural areas across the continent of Africa (ANU Forestry 2001; Gouwakinnou et al. 2011) with neither bitterness nor unpleasant odour being reported. Notwithstanding, this may be related to extensive lipid peroxidation of MSC as indicated by the high peroxide value (Table 6). This is corroborated by the absence of PUFAs in MSC (Table 3) which contrasts previous studies that found the oil in marula seed kernels to contain 5.9% linoleic acid and traces of linolenic acid (Mariod 2005). It would appear that all the linoleic and linolenic acids that might have been present in the marula kernels were completely oxidised during MSC processing or thereafter, resulting in a product with a high peroxide value. Lipid peroxidation results in stale, rancid flavour in food (Kerler and Grosch 1996), and feeding of oxidised lipids has been demonstrated to decrease body weight gain (Enberg et al. 1996), cause poor feed conversion efficiency (Cabel et al. 1988; McGill et al. 2011) and reduce meat quality (Racanicci et al. 2008; Delles et al. 2014) in broilers. Thus, consumption of oxidised lipids in MSC might have increased the absorption of lipid-derived radicals into the bloodstream, propagating oxidative reactions throughout various tissues of the birds, thereby impairing their productive performance and carcass characteristics.

**Table 6:** Oxidative and mycotoxin status of MSC

Item	Amount
Oxidative status	
Peroxide value (mEq/kg)	164.02
Thiobarbituric acid (mg/kg)	0.15
Mycotoxins (ppb)	
Aflatoxin B_1_	ND
Aflatoxin B_2_	ND
Aflatoxin G_1_	ND
Aflatoxin G_2_	ND
Deoxynivalenol	66.31
T-2 toxin	68.75
HT-2 toxin	ND
Ochratoxin A	ND
Zearalenone	ND
Fumonisin B_1_	ND
Fumonisin B_2_	ND

*ND* not detected

Another plausible mechanism underlying the deleterious effects of MSC entails infestation of this protein-rich product with mycotoxins. In this regard, we found MSC to be infested with DON and T-2 toxin, though at low levels (Table 6). Other mycotoxins might have been present in MSC; however, their levels might have been too low to be detected by analytical methods used in our study. Prolonged dietary exposure of animals to DON generally induces impaired feed intake, decreased weight gain and altered immune function (EFSA 2004). On the other hand, T-2 toxin is a highly toxic trichothecene that primarily affects the immune and haematopoietic systems (Fink-Gremmels 1999). It can also cause weight loss, liver damage, reproductive toxicity and neurotoxicity (Schuhmacher-Wolz et al. 2010). DON and T-2 toxin induce free radical production that serves as the mechanism underlying membrane and DNA damage during oxidative stress caused by these toxins (Wu et al. 2014). DON is also a well-known inhibitor of protein synthesis. It binds to peptidyl transferase (Feinberg and McLaughlin 1989), inhibiting the synthesis of RNA and DNA via binding to the ribosome. Cells and tissues with high protein turnover rates, such as the small intestine, the liver and the immune system, are most severely affected by DON intoxication (Doll et al. 2003). Although both DON and T-2 toxin were detected at rather low levels in MSC, their effects on broiler performance and carcass characteristics would appear to have been more pronounced when these toxins were present in combination and during prolonged exposure to them. Indeed, Kubena et al. (1989) found that total body weight gains and final body weights in broiler chicks were significantly reduced by the DON/T-2 toxin combination but were not significantly affected by the toxins singly. Also, whilst the incidence and severity of oral lesions induced by T-2 toxin were increased in the DON/T-2 toxin combination, several parameters not altered by DON or T-2 toxin singly were significantly affected by the combination (Kubena et al. 1989), indicating that the combination may pose a potentially greater problem to the poultry industry than either of the mycotoxins individually.

In conclusion, our results demonstrate MSC to be rich in CP, EE, Ca, P, essential (except for lysine) and non-essential amino acids, as well as OA. However, its potential use in broiler diets is limited by its rancidity and presence of mycotoxins. The use of dietary antioxidant additives and mycotoxin binders when preparing feeds may lead to improvement of the nutritional value of MSC for poultry in Africa and beyond.
